# Exploring the Antivirulence Activity of Pulverulentone A, a Phloroglucinol-Derivative from *Callistemon citrinus* Leaf Extract, against Multi-Drug Resistant *Pseudomonas aeruginosa*

**DOI:** 10.3390/antibiotics10080907

**Published:** 2021-07-25

**Authors:** Maha M. Ismail, Mariam Hassan, Sawsan S. Moawad, Mona M. Okba, Rehab M. Ashour, Nesrin M. Fayek, Fatema R. Saber

**Affiliations:** 1 Microbiology and Immunology Department, Faculty of Pharmacy, Cairo University, Cairo 11562, Egypt; mariam.hassan@pharma.cu.edu.eg; 2 Department of Pests and Plant Protection, National Research Center (NRC), Giza 12622, Egypt; ss.moawad@nrc.sci.eg; 3 Pharmacognosy Department, Faculty of Pharmacy, Cairo University, Cairo 11562, Egypt; mona.morad@pharma.cu.edu.eg (M.M.O.); rehab.ashour@pharma.cu.edu.eg (R.M.A.); nesrin.fayek@pharma.cu.edu.eg (N.M.F.)

**Keywords:** *Pseudomonas aeruginosa*, Pulverulentone A, myrtaceae, phloroglucinols, virulence, biofilm, pyocyanin, docking, *Galleria mellonella*

## Abstract

(1) Background: Bacterial resistance to antibiotics is a global life-threatening issue. Antivirulence therapy is a promising approach to combat bacterial infections as it disarms the bacteria from their virulence factors with reduced selective pressure and a lower chance of resistance. (2) Methods: *Callistemon citrinus* leaf extract and its major constituent, Pulverulentone A, were tested for their ability to inhibit biofilm, exopolysaccharides, pyocyanin and proteases produced by MDR *P. aeruginosa*. In addition, a *Galleria mellonella* larvae model was employed to evaluate the in vivo cytotoxicity of Pulverulentone A and its ability to combat *Pseudomonas* infection. Docking study was further performed to investigate Pulverulentone A druggability against main quorum sensing (QS) targets expressed by *P. aeruginosa*; (3) Results: Both *C. citrinus* extract and the isolated compound could inhibit biofilm formation, extracellular polymeric substances (EPS) and pigment production by the tested isolates. Unexpectedly, no significant inhibition was observed on proteases production. The in silico docking analysis revealed good interactions of Pulverulentone A with all QS targets examined (LasR, MyfR/PqsR, QscR). Pulverulentone A was safe up to 400 µg·mL^−1^ in *Galleria* caterpillars. Moreover, *pre*-treatment of *P. aeruginosa* with Pulverulentone A slightly enhanced the survival of the infected larvae. (4) Conclusions: The present study proves Pulverulentone A safety with significant in vitro and in silico antivirulence potential against *P. aeruginosa*.

## 1. Introduction

*Pseudomonas aeruginosa* is considered one of the serious opportunistic bacteria responsible for many life-threatening conditions and hospital-acquired infections globally, including sepsis, urinary tract implant infection and burn and wound infections [1]. It forms biofilms on both biotic and abiotic surfaces which confer resistance to most of the available antibiotics [2]. There were about 32,600 estimated MDR *P. aeruginosa* infections in hospitalized patients, in addition to 2700 estimated deaths due to this infection, according to what was reported in the USA in 2017 [3]. Carbapenem-resistant *P. aeruginosa* has been listed by WHO as one of the critical, high-priority pathogens requiring much attention for the discovery and development of novel antimicrobials [4]. Diverse virulence factors of *P. aeruginosa* support its ability to cause diseases and microbial resistance; these include the ability to form biofilm which hinders penetration of antimicrobials, the antioxidant pyocyanin pigment which plays an important role in bacterial iron uptake and metabolism, and controls efflux pump genes and monooxygenase genes. Pyocyanin is one of the most important virulence factors as it is capable of inhibiting cell respiration and ciliary movement, leading to the reduction in mucus secretion during airway infections [5].

The need for antivirulence agents and bacterial biofilm inhibitors rather than antimicrobials from natural sources is deemed necessary for applying phytotherapy [6]. Their underlying mechanisms involve quorum sensing (QS) inhibition, disruption of the polymeric biofilm matrix and decrease in the associated virulence factors; these render the bacteria powerless and defenseless with a slighter chance of resistance as a result of the reduced selective pressure exerted on the bacteria [7,8,9].

There are three well-identified QS systems in *P. aeruginosa*, Las, Rhl and PQS, and these systems mediate the synthesis of 3-oxo-C12-HSL, C4-HSL and PQS, respectively. Rhl and Pqs are hierarchically mediated by Las. The molecular targets, autoinducers and transcription factors (LasR, QscR, RhlR and MvfR/PqsR) of these QS systems have been well studied [10,11]. These QS systems are considered promising drug targets, and they are essential for expression of virulence factors such as pyocyanin and proteases in addition to biofilm formation. Mutations in these systems inhibited pathogenicity of *P. aeruginosa* [12]. In silico approaches to assess structure-based druggability of a QS system have aided in the development of effective antivirulence agents against bacteria [13].

Previous reports investigated the potential of natural products against several biofilm-forming pathogens [14,15]. *Citrus* limonoids, as exemplified by isolimonic acid and hordenine, affected signaling pathways in bacterial cells [16]. Moreover, phenolic compounds, as represented by flavonoids, i.e., quercetin and kaempferol, exhibited a pronounced biofilm inhibition of *S. mutans* and thus were recommended as anti-caries agents.

In the same context, the plant family Myrtaceae exhibits a complex phytochemical make-up with an array of potent bioactivities, where flavonoids, phenolic acids, triterpenes, volatile oils and phloroglucinols together with their corresponding adducts are the most prevalent representatives [17,18,19,20]. Myrtenol, a constituent of *Myrtus communis* essential oil, inhibited the biofilm formation of *S. aureus* at sub-inhibitory concentration, which was further verified by molecular docking [14].

Previous studies have highlighted the antimicrobial activities of essential oils and extracts of *Callistemon citrinus* [21,22]. In addition, callistemenonone A, a dimeric phloroglucinol isolated from *Callistemon viminalis,* showed a potent bactericidal action against MRSA [23]. Nevertheless, further studies to explore the antimicrobial and antivirulence potential of bioactive constituents from *C. citrinus* are still needed for better exploitation of this traditionally used plant.

As a part of our continued research on myrtaceous plants as possible bacterial biofilm inhibitors [6,24], *Callistemon citrinus* leaves have been investigated for bioactive phytoconstituents. Accordingly, the major phloroglucinol compound, Pulverulentone A, previously isolated from *C. citrinus* extract, has been subjected to detailed microbiological assessment to pinpoint its underlying mechanism against some *P. aeruginosa* virulence factors and biofilms.

## 2. Results and Discussion

The global spread of microbial resistance makes it urgent to find an alternative way to control infections [25]. Antivirulence therapy involves the attenuation of microbial virulence factors and hence microbial pathogenicity, enhancing the clearance of the infection by the immune system [26].

The aim of this study was to investigate the antivirulence activity of *Callistemon citrinus* extract and its major isolated constituent, Pulverulentone A, against MDR *P. aeruginosa* isolates.

The virulence of 10 clinical and environmental isolates of *P. aeruginosa* was screened by assessing the ability of these isolates to form biofilm and to produce pyocyanin pigment. Screening the biofilm formation ability of the isolates revealed that four isolates (PA-1, PA-3, PA-5 and PA-7) showed the ability to form strong biofilm, five isolates (PA-2, PA-4, PA-6, PA-9 and PA-10,) were capable of forming weak biofilm and one isolate (PA-8) showed negative biofilm formation. Among the screened isolates, strain PA-7 showed the highest degree of pyocyanin pigmentation, producing 7.21 ± 0.64 µg·mL^−1^ of pyocyanin while PA-3 and PA-10 strains showed moderate pigmentation, producing 4.26 ± 1.17 and 3.03 ± 1.22 µg·mL^−1^ pyocyanin, respectively. No statistically significant difference exists between the concentration of pyocyanin produced by strain PA-7 and PA-3 (one-way ANOVA, Dunnett’s multiple comparisons test, *p* = 0.0094). Other isolates produced pyocyanin in concentrations ranging from 0.65 to 0.09 µg·mL^−1^ and were regarded as negative pigment producers. The same strains, PA-7 and PA-3, showed the ability to form strong biofilm, with an average BF of 0.47 and 0.62, respectively, while isolate PA-10 showed weak biofilm formation capability. Consequently, *P. aeruginosa* isolates PA-7 and PA-3 were chosen for further experiments.

**Figure 1:**
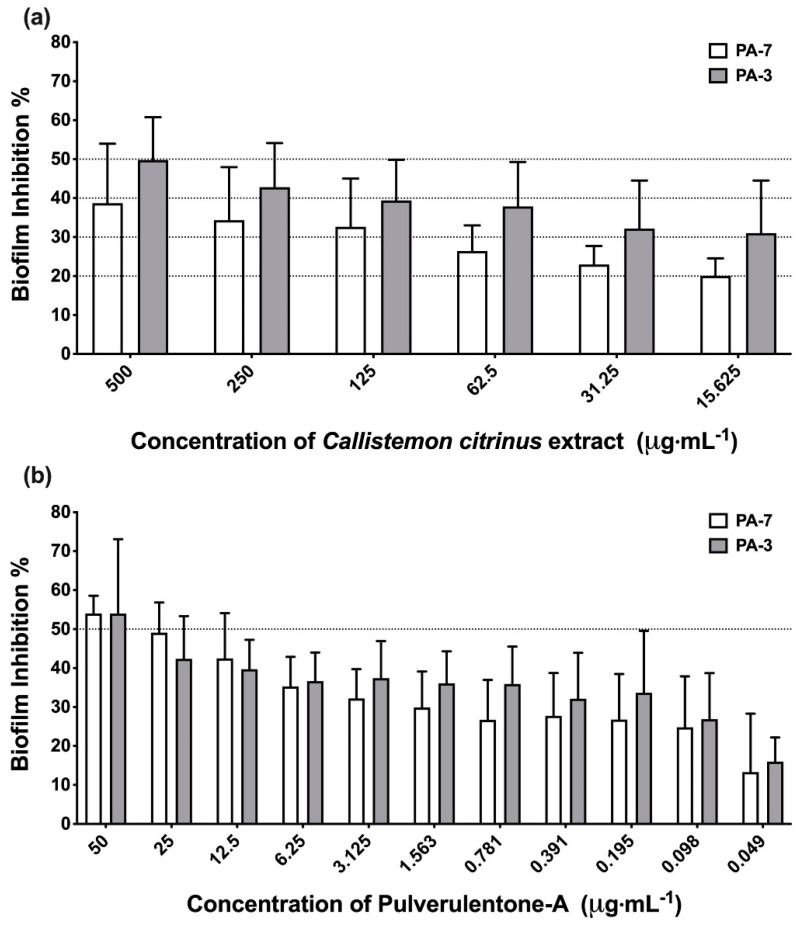
(**a**) Biofilm inhibition activity of *Callistemon citrinus* extract against *P. aeruginosa* PA-7 and PA-3 at concentrations ranging 500–15.625 µg·mL^−1^. (**b**) Biofilm inhibition activity of Pulverulentone A against *P. aeruginosa* PA-7 and PA-3 at concentrations ranging 50–0.049 µg·mL^−1^. Data represent the means of biofilm inhibition percentages ± SD, *n* = 3. A statistically significant difference exists between the effect of the tested concentrations of extract/Pulverulentone A (two-way ANOVA, *p* < 0.005).

The methylene chloride-methanol extract of *C. citrinus* and the isolated compound Pulverulentone A inhibited the biofilm formation of PA-7 and PA-3 strains in a dose-dependent manner (Figure 1). The tested concentrations of *C. citrinus* extract and Pulverulentone A significantly inhibited biofilm formation (two-way ANOVA, *p* < 0.005 and *p* < 0.0001, respectively). The *C. citrinus* extract showed 39% ± 8.8% and 50% ± 6.3% inhibition of biofilm formed by strains PA-7 and PA-3, respectively, at a concentration of 500 µg·mL^−1^. In addition, Pulverulentone A showed 54% ± 2.6% and 54% ± 11% inhibition of biofilm formed by strains PA-7 and PA-3, respectively, at a concentration of 50 µg·mL^−1^. There was no significant difference between the biofilm inhibition activity of *C. citrinus* against the two strains at all the tested concentrations (two-way ANOVA, Sidak’s post-test, *p* < 0.05). The same findings were recorded for the biofilm inhibition activity of Pulverulentone A.

**Figure 2:**
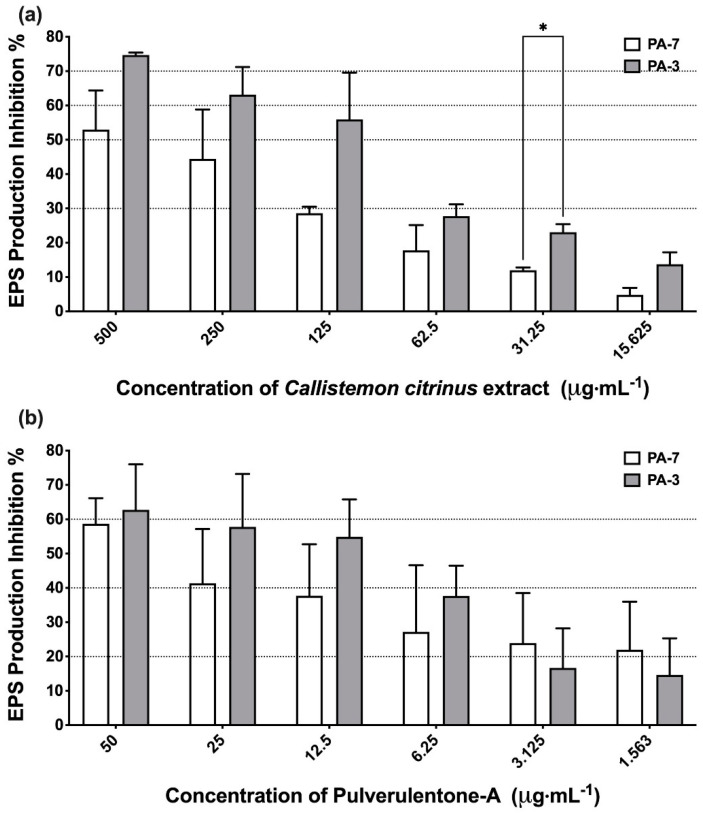
(**a**) Inhibition percentage of extracellular polymeric substances (EPS) production by *Callistemon citrinus* extract against *P. aeruginosa* PA-7 and PA-3 at concentrations ranging 500–15.625 µg·mL^−1^. (**b**) Inhibition percentage of extracellular polymeric substances (EPS) production by Pulverulentone A against *P. aeruginosa* PA-7 and PA-3 at concentrations ranging 50–1.563 µg·mL^−1^. Data represent the means of EPS production inhibition percentage ± SD, *n* = 3. A statistically significant difference exists between the effect of the tested concentrations of extract/Pulverulentone A (two-way ANOVA, *p* < 0.0001). * Means that the difference is significant at *p* < 0.05 (two-way ANOVA, Sidak’s post-test).

The extracellular polymeric substances (EPS) are considered the major components in the establishment of biofilm systems. Hence, *C. citrinus* extract and Pulverulentone A were assessed for their ability to inhibit the production of EPS (measured as polysacchairdes) by PA-7 and PA-3 strains (Figure 2). *C. citrinus* extract and Pulverulentone A both could significantly inhibit EPS production by both strains at the tested concentrations (two-way ANOVA, *p* < 0.0001). The *C. citrinus* extract showed high inhibition percentage of EPS production by strains PA-7 and PA-3, up to 53% ± 6.6% and 75% ± 0.4%, respectively, at a concentration of 500 µg·mL^−1^ (Figure 2a). The same pattern of EPS production inhibition percentage was observed with Pulverulentone A, with inhibition up to 59% ± 7.4% and 63% ± 13.3% for strains PA-7 and PA-3, respectively, at a concentration of 50 µg·mL^−1^ (Figure 2b). There was no significant difference between EPS production inhibition activity of *C. citrinus* extract/ Pulverulentone A against the two strains at the all tested concentrations except for *C. citrinus* extract at a concentration of 31.25 µg·mL^−1^ (two-way ANOVA, Sidak’s post-test, *p* = 0.0496).

**Figure 3:**
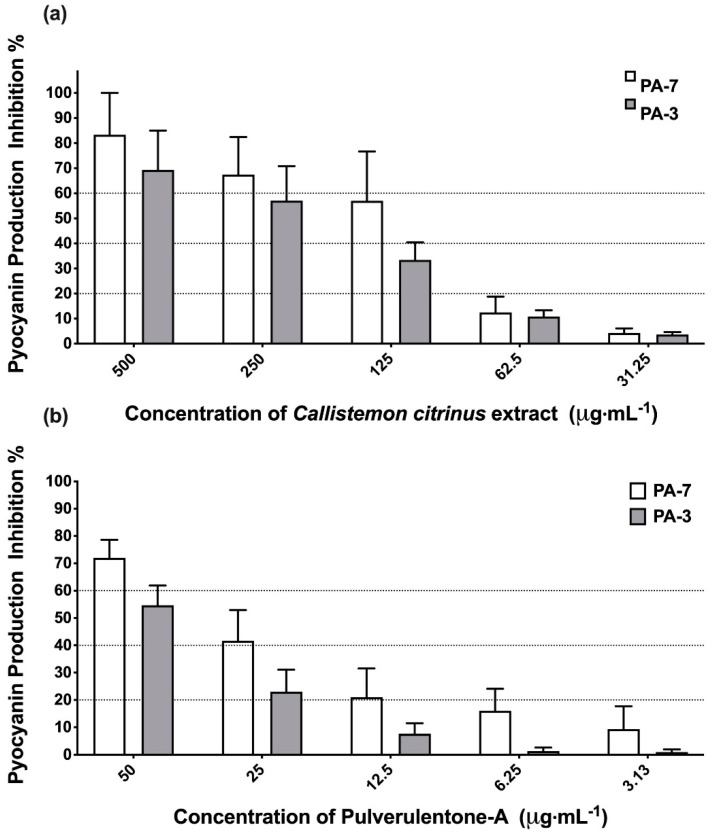
(**a**) Inhibition percentage of pyocyanin production by *Callistemon citrinus* extract against *P. aeruginosa* PA-7 and PA-3 at concentrations ranging 500–125 µg·mL^−1^. (**b**) Inhibition percentage of pyocyanin production by Pulverulentone-A against PA-7 and PA-3 at concentrations ranging 50–3.125 µg·mL^−1^. Data represent the means of pyocyanin production inhibition percentage ± SD, *n* = 3. A statistically significant difference exists between the effect of the tested concentrations of Pulverulentone A (two-way ANOVA, *p* < 0.0001).

Pyocyanin, a bluish-green redox-active secondary metabolite, plays a crucial role in the virulence of *P. aeruginosa* during infection [27]. It has been reported that the production of pyocyanin by *P. aeruginosa* has adverse effects on the central nervous system, cardiovascular system, respiratory system and urological system [27]. Accordingly, *C. citrinus* extract and Pulverulentone A were evaluated for their ability to inhibit the production of pyocyanin by PA-7 and PA-3 strains (Figure 3). *C. citrinus* extract showed inhibition to pyocyanin production up to 83% ± 16.6% and 69% ± 15.6% by strains PA-7 and PA-3, respectively, at 500 µg·mL^−1^ (Figure 3a). The same pattern of pyocyanin production inhibition was observed with Pulverulentone A (72% ± 6.6% and 55% ± 7.2% for strains PA-7 and PA-3, respectively) at a concentration of 50 µg·mL^−1^ (Figure 3b).

**Figure 4:**
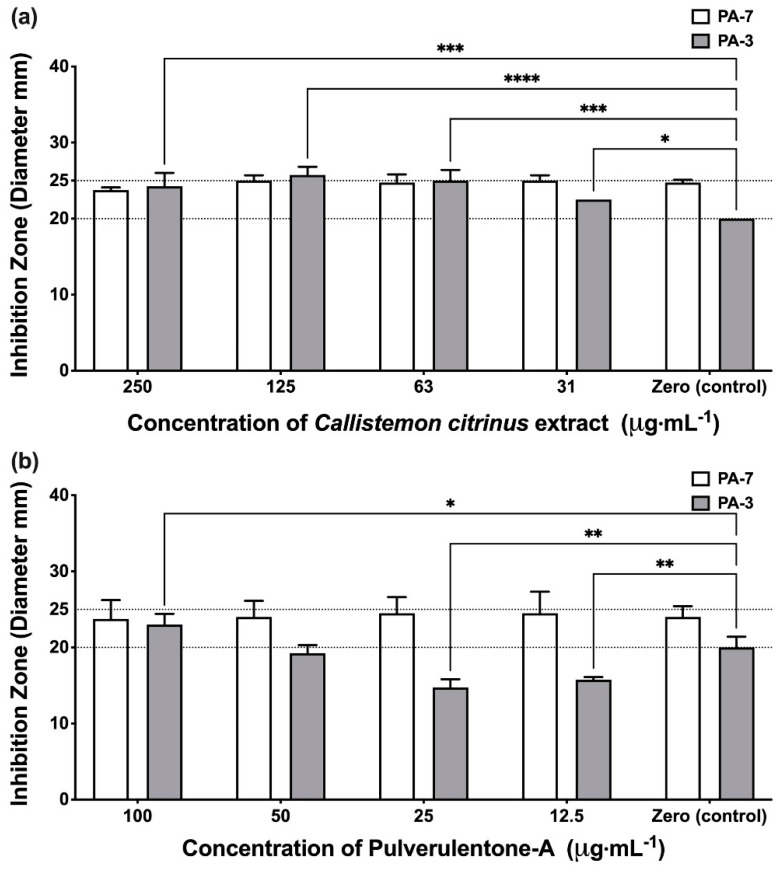
(**a**) Proteases production inhibition activity of *Callistemon citrinus* extract against *P. aeruginosa* PA-7 and PA-3 at concentrations ranging 250–31 µg·mL^−1^. (**b**) Proteases production inhibition activity of Pulverulentone A against *P. aeruginosa* PA-7 and PA-3 at concentrations ranging 100–12.5 µg·mL^−1^. Data represent the means ± SD, *n* = 3. A statistically significant difference exists between the effect of the tested concentrations of extract/Pulverulentone A (two-way ANOVA, *p* < 0.05). *, **, *** and **** mean that the difference is significant at *p* < 0.05, 0.008, 0.0005 and 0.0001, respectively (two-way ANOVA, Dunnett’s post-test).

Protease enzyme is one of the most important bacterial virulence factors predisposing to cystic fibrosis and wound infections [28]. The results showed that the effect of the *C. citrinus* extract and Pulverulentone A on the proteolytic activity was isolate-dependent. Unexpectedly, neither the extract nor Pulverulentone A showed any significant inhibitory effect against proteolytic activity of isolate PA-7 (two-way ANOVA, Dunnett’s post-test, *p* < 0.05) (Figure 4a,b); this was unlike what was observed against biofilm, EPS and pigment production. Interestingly, the treatment of isolate PA-3 with *C. citrinus* extract (at all the tested concentrations) showed significantly higher proteolytic activity when compared to the untreated control (two-way ANOVA, Dunnett’s post-test, *p* < 0.05) (Figure 4a). This may be attributed to the presence of a mixture of different compounds in the *C. citrinus* extract that could interact and enhance the production of proteases by isolate PA-3. The only proteolytic inhibitory activity was observed when isolate PA-3 was treated with Pulverulentone A at concentrations of 12.5 and 25 µg·mL^−1^ (two-way ANOVA, Dunnett’s post-test, *p* < 0.008) (Figure 4b).

Although the activity of *C. citrinus* extract and Pulverulentone A on biofilm formation (Figure 1) and EPS production (Figure 2) was higher against PA-3 than against PA-7 (with no significant difference, two-way ANOVA, Sidak’s post-test, *p* < 0.05), their inhibitory activities on pyocyanin production (Figure 3) were higher against PA-7 than PA-3 strains (non-significant difference, two-way ANOVA, Sidak’s post-test, *p* < 0.05).

The anti-biofilm and anti-pigment production activities observed for both the extract and Pulverulentone A are in agreement with previous findings [24]. Pulverulentone A exhibited higher anti-biofilm activity than the crude *C. citrinus* extract against MRSA and MSSA biofilms, resulting in 71% and 62% inhibition, respectively. Moreover, the same compound displayed the highest degree of staphyloxanthin pigment production inhibition by MRSA and MSSA by an average of ≈55% among the extract and the other isolated compounds, 8-desmethyl eucalyptin and eucalyptin.

Several phloroglucinol compounds proved their wide-range inhibition effectiveness against the growth of bacteria and its biofilm formation, such as compounds previously isolated from *Hypericum* spp [29]. Interestingly, the plant-beneficial microorganisms such as *Pseudomonas* spp. produce several phloroglucinol derivatives such as 2,4-diacetylphloroglucinol, commonly recognized as secondary metabolites with an important role as antibiotics via signaling molecules and as pathogenicity factors [30]. In addition, these phloroglucinol derivatives were recently regarded as effective bio-control agents that provide protection for the plant root system from numerous soil-borne plant diseases [30].

In general, phytochemicals and plant-derived substances are well known for their antimicrobial and antivirulence activities. El-Sayed and co-workers [31] studied the effect of some plant extracts (ethanolic extracts of olive leaf and green tea) on the virulence-associated traits of the same strains of *P. aeruginosa* employed in this study. They reported *P. aeruginosa* QS genes to undergo down-regulation upon treatment with olive leaf and green tea extracts. Additionally, strong inhibition of biofilm, twitching motility and pyocyanin production was also observed. Similar activity against *P. aeruginosa* PAO1 was observed in another study [32] by hordenine—a major phenolic dietary compound from sprouting parsley extract—at concentrations of 0.5 to 1.0 mg·mL^−1^. Moreover, hordenine, at the same tested concentrations, could increase the susceptibility of *P. aeruginosa* PAO1 biofilm to a sub-MIC level of an aminoglycoside antibiotic, netilmicin (0.4 µg·mL^−1^). This combination has led to biofilm formation reduction up to 88%. Hordenine could significantly enhance the effect of netilmicin on *P. aeruginosa* PAO1 biofilms in a concentration-dependent manner, while sole application of netilmicin showed negligible biofilm reduction.

Froes and co-workers isolated calycopterin, a major flavonoid from the ethanolic extract of *Marcetia latifolia* that was tested against *P. aeruginosa* ATCC 27853 [33], where an inhibitory effect on pyocyanin production by 77.4% at a concentration of 267.3 µM and reduction in the swarming motility in a dose-dependent manner were observed. On the contrary, calycopterin enhanced biofilm formation by the tested strain, which was attributed to the probability of calycopterin to favor the sessile lifestyle of bacteria by increasing c-di-GMP levels, leading to reduction in pyocyanin production and flagellar activity; as a consequence, biofilm formation was enhanced.

In order to better visualize the possible interactions of Pulverulentone A with the target proteins of *Pseudomonas aueroginosa*, in silico study has been adopted. Hence, the binding modes and affinities of Pulverulentone A within the binding sites of LasR, MyfR and QscR were examined using molecular docking.

Docking setup was first validated by self-docking of the co-crystalized ligands TY4, CZG and EVY in the vicinity of the binding site of the enzymes LasR, MyfR and QscR, respectively.

**Figure 5:**
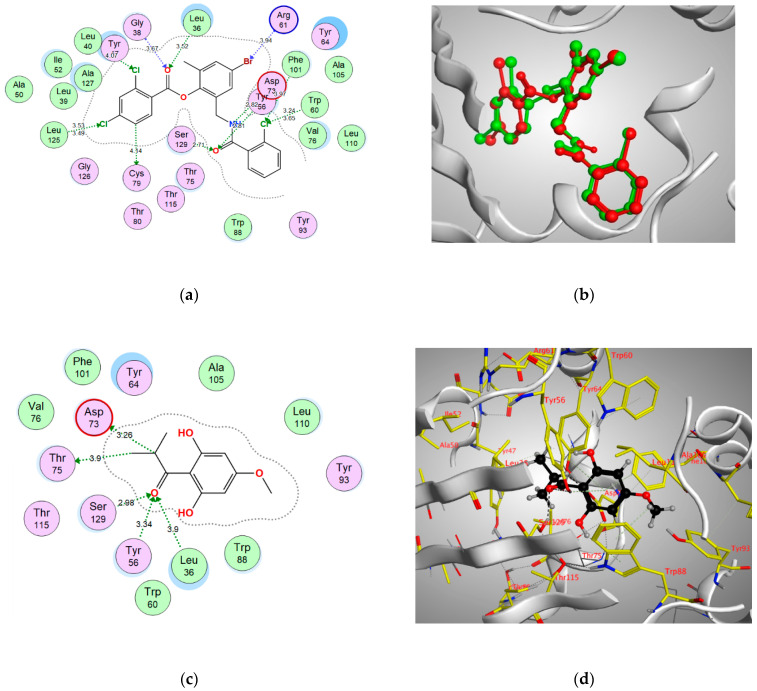
(**a**) Two-dimensional interactions of the co-crystallized ligand TY4 within LasR active site, (**b**) 3D representation of the superimposition of the co-crystallized (red) and the docking pose (green) of CZG in the active site of MvfR/PqsR. (**c**,**d**) 2D and 3D diagrams of pulverulentone A interactions within MvfR/PqsR binding site.

**Figure 6:**
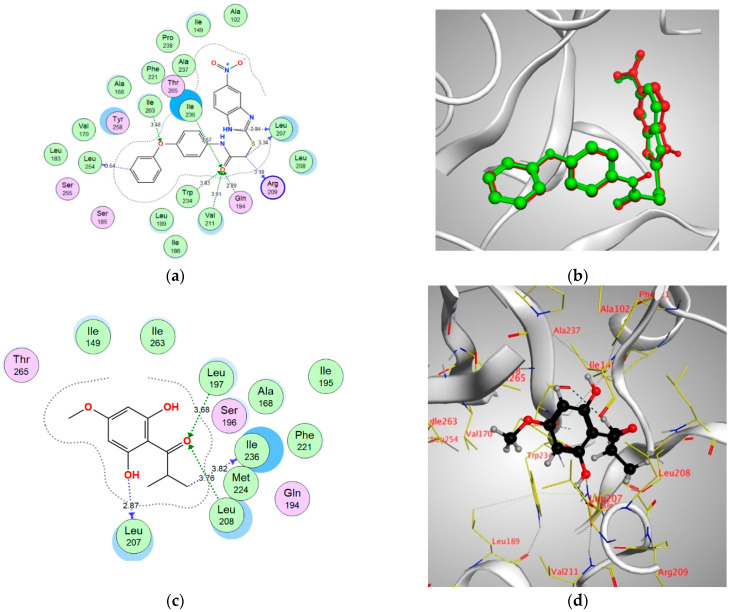
(**a**) Two-dimensional interactions of CZG within MvfR/PqsR active site, (**b**) 3D representation of the superimposition of the co-crystallized (red) and the docking pose (green) of CZG in the active site of MvfR/PqsR, (**c**,**d**) 2D and 3D diagrams of Pulverulentone A interactions within MvfR/PqsR binding site.

**Figure 7:**
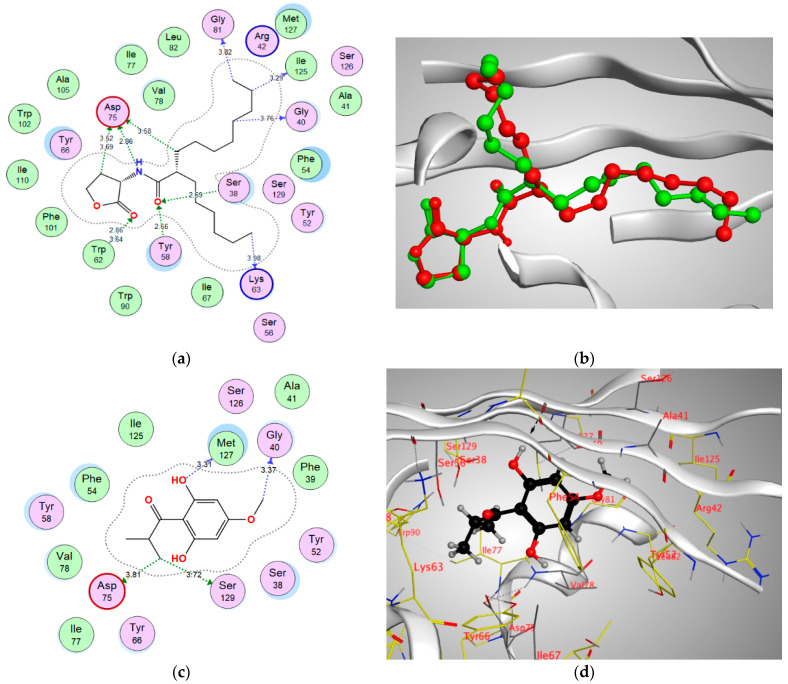
(**a**) Two-dimensional interactions of EVY within QscR active site, (**b**) 3D representation of the superimposition of the co-crystallized (red) and the docking pose (green) of EVY in the active site of QscR, (**c**,**d**) 2D and 3D diagrams of Pulverulentone A interactions within QscR binding site.

TY4 binding with LasR showed a docking score (S) of −16.8177 kcal/mol and root mean square deviation (RMSD) 0.7570 Å. In addition, TY4 showed strong bond interactions with Leu36, Gly38, Tyr47, Tyr56, Trp60, Arg61, Asp73, Cys79, Leu125 and Ser129 in the enzyme binding sites. Moreover, CZG showed binding with MyfR enzyme with a docking score of −13.4171 kcal/mol. and RMSD of 0.2396 Å and there was a strong bond interaction with Gln194, Leu207, Arg209, Val211, Trp234, Ile236, Leu254 and Ile263. In addition, the docking score of ligand EVY binding within the active site of QscR was −16.1479 kcal/mol. and RMSD was 0.6476 Å. In addition, EVY had strong bond interactions with Ser38, Gly40, Tyr58, Trp62, Lys63, Asp75, Gly81 and Ile125 (Figure 5, Figure 6 and Figure 7).

Pulverulentone A showed good energy binding scores and binding interactions with various amino acids within the active sites of the three examined enzymes (LasR, MyfR and QscR) by both hydrogen bonding and hydrophobic interaction (Table 1). It was noticed that O (C=O) and O CH_3_ groups of Pulverulentone A are the interacting groups with LasR, MvfR/PqsR and QscR (Table 1). These outcomes come in accordance with previous results that revealed the pronounced antibacterial effect in parallel with the presence of Pulverulentone A O-methylation and highlighted how much this activity is directly correlated with the acyl side [34,35]. The scores and interactions were inferior to that of the co-crystalized ligands, and this can be attributed to the small size of Pulverulentone A relative to the size of the co-crystallized ligands (Figure 5, Figure 6 and Figure 7); however, it can still interfere with the binding of the natural autoinducers to its receptor, leading to a considerable inhibition of quorum sensing and virulence, as was confirmed by the in vitro biofilm formation, EPS production and pyocyanin inhibition. To the best of our knowledge, this is the first study to examine the druggability of Pulverulentone A against *P. aeruginosa* QS system enzymes.

Previous studies have examined various compounds of natural and synthetic origin as anti-QS drugs against *P. aeruginosa* and showed results comparable to this study. Zhou et al. [7] investigated the probable binding affinities of the phenolic compound hordenine to the LasR, RhlR and PqsR ligand-binding domains using More2014 module. They observed that the binding affinity of the native autoinducer 3-oxo-C12-HSL with LasR was stronger than the binding of hordenine to LasR active site (−11.62 and −6.72 kcal/mol, respectively). This is in line with this study, where the binding affinity observed by the co-crystalized ligand TY4 with LasR was stronger than that observed for Pulverulentone A to bind LasR. The same compound (hordenine) showed strong binding affinities to both RhlR and MvfR/PqsR equivalent to that obtained from the interaction of the native ligand to theses enzymes (−5.5 and −6.6 kcal/mol, respectively), which is superior to the binding results of Pulverulentone A to MvfR/PqsR active site obtained in this study.

Furthermore, Abbas et al. [36] reported the antidiabetic drug, sitagliptin, to have anti-QS and antivirulence activity against *P. aeruginosa* PAO1 strain at sub-MIC level. It could inhibit production of various virulence factors such as proteases, pyocyanin and hemolysin by 38%, 62% and 92%, respectively. In addition, blocking the swimming, swarming and twitching motilities by 47%, 75% and 77%, respectively, and biofilm inhibition by 54.7% were recorded. Sitagliptin also interfered with binding of the natural autoinducers with LasR enzyme. Moreover, a reduction in expression of QS genes has been detected, and thus these authors recommended sitagliptin for the treatment of *P. aeruginosa* systemic infections in diabetic patients or topical infections in non-diabetic patients.

The cytotoxicity of Pulverulentone A performed using *G. mellonella* larvae showed that it was safe at all the tested concentrations up to 400 µg·mL^−1^ (the highest tested concentration). This indicates its suitability for in vivo applications at this concentration range. Caterpillars of the greater wax moth *G. mellonella* are considered an alternative promising popular model to investigate the efficacy and toxicity of new antimicrobial and antivirulence compounds. Jander et al. [37] reported a significant positive correlation between the virulence of *P. aeruginosa* in *G. mellonella* and mice. This indicates the suitability of this insect model to study bacterial pathogenicity and virulence patterns the same as using mice models. Khalil et al. [38] have employed the *G. mellonella* model to investigate the virulence pattern of carbapenem-resistant strains of *K. pneumonia*. Another study evaluated the efficacy of a combination of colistin and cotrimoxazole against carbapenem-resistant *A. baumannii*, applying the same insect model [39].

Moreover, being an invertebrate, the *G. mellonella* insect offers a cheaper and simpler model to provide data prior to mammalian studies. In addition, they are affected by the same virulence factors required to infect mammals, and their immune system is identical to mammals, as well [40].

A previous study by Allegra et al. [41] examined and compared the in vivo cytotoxicity testing of low toxicity chemicals using a *G. mellonella* larvae model and cell culture systems, and it was proven that *G. mellonella* larvae is a more reliable means for toxicity prediction of low toxicity chemicals than cell culture systems, which overrated their toxicity.

Infection of *G. mellonella* larvae with PA-7 strain pretreated with either Pulverulentone A at 100 µg·mL^−1^ or sub-MIC of gentamycin (250 µg·mL^−1^) was performed to determine the ability of the isolated compound to enhance the survival of infected larvae. Based on the observed results of the in vitro antivirulence activities of Pulverulentone A, it was hypothesized that it can also prevent a lethal infection in a eukaryotic host. Negative control groups (PBS-injected and quality control group) showed 100% survival during the experiment.

The survival of the infected caterpillars was slightly enhanced by pretreatment of the bacteria with Pulverulentone A (13% survival on day 4) compared to pretreatment with sub-MIC of gentamycin (7% survival on day 4). This difference was found to be non-significant (log-rank (Mantel–Cox) test, *p* = 0.3034).

**Figure 8:**
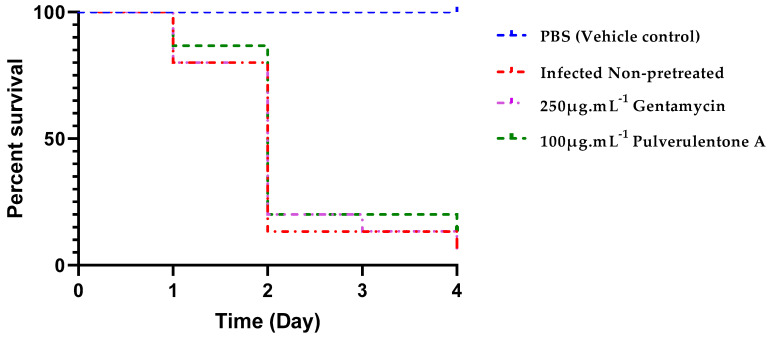
Percent survival of *G. mellonella* larvae infected with *P. aeruginosae*-7 strain within 4 d. PA-7 cells were pretreated with 100 µg·mL^−1^ Pulverulentone A or 250 µg·mL^−1^ gentamycin overnight before infection. Positive control (larvae infected with non-pretreated cells) and negative controls (larvae injected with PBS (vehicle) and larvae non injected (quality control)) were included. Log-rank (Mantel–Cox) test, *p* < 0.0001, *n* = 15.

The group of larvae infected with non-pretreated *P. aeruginosa* exhibited 93% death on day 4, similar to the group infected with gentamycin-pretreated *P. aeruginosa* (Figure 8). No previous studies on the in vivo antivirulence activity of Pulverulentone A were reported. In a study performed by Tharmalingam et al. [42], a new compound named BIP (4-(1,3-dimethyl-2,3-dihydro-1H-benzimidazol-2-yl)phenol) was identified during high throughput screening for antivirulence compounds against MRSA. These authors reported this compound to prolong the survival of MRSA-infected *G. mellonella* within 24 h. It is worthy to note that both BIP and Pulverulentone A are phenolic compounds.

To the best of our knowledge, this is the first study exploring the in vitro, in vivo and in silico potential of Pulverulentone A as a *Pseudomonas aeruginosa* anti-virulent drug candidate. Our findings recommend further studies on different phloroglucinols and phloroglucinols adducts for the purpose of discovering novel antivirulence hits.

## 3. Materials and Methods

### 3.1. Plant Material

Leaves of *Callistemon citrinus* Skeels were collected from Orman Botanical Garden, Giza, Egypt. The identity of the plant material was confirmed by Therese Labib, botanical specialist and consultant at Orman and Qubba Botanical Gardens. A voucher specimen (1.07.2019/1) was deposited at Pharmacognosy Department, Faculty of Pharmacy, Cairo University.

### 3.2. Preparation of the Extract and Isolation of Pulverulentone A

Details of the preparation of the methylene chloride-methanol extract (80:20) of *C. citrinus* leaves, isolation of Pulverulentone A and NMR data of the compound have been previously described [24]. In brief, the air-dried leaves of *Callistemon citrinus* were extracted in a Soxhlet apparatus (Glassco, Haryana, India) using methylene chloride:methanol (80:20). All chemicals used are of analytical grade. The crude extract was then applied on a VLC column of Silica Gel H (E-Merck, Darmstadt, Germany) and eluted using gradient system of hexane:ethyl acetate (100:0 to 0:100) followed by CH_2_Cl_2_/CH_3_OH (100:0 until 20% methanol). Fractions were monitored by TLC using different solvent systems: hexane-ethyl acetate (95:5 *v*/*v*), hexane-ethyl acetate (85:15 *v*/*v*), methylene chloride-methanol (95:5 *v*/*v*) and methylene chloride-methanol (85:15 *v*/*v*). Similar fractions were pooled together. For compounds’ visualization on TLC, natural product/polyethylene glycol (NP/PEG) and *p*-anisaldehyde sulphuric acid were used as spray reagents in addition to UV lamp (254 & 365 nm) in case of NP/PEG. Consequently, the major fraction II was purified on successive preparative RP-18 TLC Glass plates (E-Merck, Darmstadt, Germany), using H_2_O:CH_3_OH (10:90) as an eluting system. After that, Rp-18 silica column was utilized and eluted with 100% methanol. Preparative RP-18 HPLC PuriFlash® column (Interchim, Montluçon, France) was finally used, applying gradient elution (H_2_O: CH_3_OH, 20:80 to 0:100) for the isolation of Pulverulentone A, which was isolated as 130 mg of white needles.

For structure elucidation of Pulverulentone A, both ^1^H and ^13^C-NMR analyses were recorded on a Bruker AVIIIHD400 FT–NMR spectrometer (400/3) instrument (Kanagawa, Japan). TMS was used as internal standard and chemical shifts were given in *δ* ppm value. Where, the obtained NMR data was in total agreement with the previous report on the methoxylated phloroglucinol compound; Pulverulentone A [43].

### 3.3. Microorganisms and Growth Conditions

Ten clinical and environmental isolates of MDR *Pseudomonas aeruginosa* were used in this study. The clinical isolates were collected in a previous study from patients in different hospitals in Cairo and Alexandria, Egypt [31]. The isolates were cryopreserved as glycerol stock. The strains were grown aerobically on Luria-Bertani (L.B) agar/broth or Tryptone soy agar/broth (TSA/TSB) (Oxoid, UK) with orbital shaking at 180 rpm at 37 °C for 24 h. All experiments were conducted in triplicates unless otherwise specified.

### 3.4. Effect of the Extract/Pulverulentone A on Biofilm Formation

#### 3.4.1. Screening for Biofilm Formation by the Tested Isolates

Initially, all *P. aeruginosa* isolates were screened for their ability to form biofilm. Each isolate was allowed to form biofilm according to the method described by [25,44] with slight modifications. Briefly, overnight culture of each test isolate was prepared in TSB according to the above mentioned growth conditions: the turbidity of each culture was adjusted to OD_600_ of 0.1–0.125, then diluted 1:100 in fresh TSB. Then, 120 µL of each of the diluted cultures were dispensed in the wells of 96-well flat-bottom microplates. After that, the plates were incubated statically at 37 °C for 24 h. After incubation, biofilm was stained and visualized as follows: the turbidity of the growth was measured at OD_600_ before discarding the spent medium and planktonic suspended cells. Wells containing the attached biofilm were washed three times with normal phosphate-buffered saline (PBS) solution, then the plates were left to dry completely. Then, 120 µL of 0.1% crystal violet solution were added to each well and plates were left static for 20 min. The plates were washed three times with de-ionized water and then left to dry completely. Then, 150 µL of absolute ethanol were added to each well for 20 min to extract the violet color of crystal violet. Then, 100 µL of the solubilized crystal violet were transferred to a new microplate to be measured at wavelength of 550 nm. The test isolates were classified as strong, moderate, weak or non-biofilm producing according to the following equation [45]:

BF=AB−CW

where BF is biofilm formation by the test isolate, AB is OD_550_ of stained bacteria cells attached to the wells and CW is OD_550_ of stained negative control wells (sterile plain medium).

A strong biofilm producing strain results in BF > 0.300 and a moderate strain shows BF = 0.200–0.299; if BF = 0.100–0.199, it is interpreted as a weak biofilm forming strain. A BF value less than 0.100 means the isolate is non-biofilm producing. Only strong or moderate biofilm forming strains were selected to perform further anti-biofilm assays.

#### 3.4.2. Biofilm Inhibition Activity of the Extract/Pulverulentone A

*C. citrinus* leaf methylene chloride-methanol extract and Pulverulentone-A were investigated for their biofilm inhibition activity at the concentrations proven not to cause growth inhibition, discoloration or precipitation when added to the TSB medium (to avoid interference with biofilm visualization). Absence of growth inhibition was assessed by measuring the optical density at OD_600_ after incubation, while medium discoloration or precipitation was assessed by visual examination. The tested concentrations ranged from 500 to 15.625 µg·mL^−1^ and 50 to 0.049 µg·mL^−1^ for the extract and Pulverulentone A, respectively. Biofilm inhibition assay was performed according to abovementioned steps in the presence of either the extract/Pulverulentone A. Both positive control (DMSO, the solvent, was added to the media instead of the *C. citrinus* extract/Pulverulentone-A) and negative control (uninoculated TSB) were employed.

### 3.5. Quantification of Extracellular Polymeric Substances Represented as Polysaccharides

In order to estimate the extracellular polymeric substances (EPS), which are composed mainly of polysaccharides in addition to nucleic acids and proteins, the procedure described by Nithya et al. [46] was followed. In brief, the relevant isolates were allowed to form biofilm under the same above mentioned conditions in the presence of either the extract or Pulverulentone A at the same mentioned concentrations. After 24 h, planktonic cells and spent medium were discarded and the attached biofilm was washed three times using PBS solution. Then, 50 µL of PBS was added to each well, and an equal volume of 5% phenol (50 µL) and 5 volumes (250 µL) of concentrated sulfuric acid were added and the mixture was incubated under darkness for 1 h. After incubation, the absorbance of the mixture was measured at 490 nm. Positive control (DMSO, the solvent, was added to the media instead of the *C. citrinus* extract/Pulverulentone-A) was included.

The inhibition % of EPS production was calculated according to this equation:

$$\mathrm{EPS}\;\mathrm{Inhibition}\;\%=\frac{{\mathrm{Positive}\;\mathrm{control}}_{\mathrm{OD}490}-{\mathrm{Test}}_{\mathrm{OD}490}}{{\mathrm{Positive}\;\mathrm{control}}_{\mathrm{OD}490}}\times 100$$

### 3.6. Effect of the Extract/Pulverulentone A on pyocyanin Pigment Production

Initially, *P. aeruginosa* isolates were screened for their ability to produce pyocyanin pigment in glycerol alanine minimal medium [47]. The optical density of the test strains was adjusted to OD_600_ of 0.1, then 200 µL of each isolate were dispensed in 96 well U-shaped microplates. Plates were incubated at 37 °C for 24 h under orbital shaking at 180 rpm. Negative control was included (uninoculated medium). The concentration of pyocyanin pigment produced was determined according to the equation:

$$\mathrm{Concentration}\;\mathrm{of}\;\mathrm{pyocyanin}\;(µg.{\mathrm{mL}}^{-1})={\mathrm{OD}}_{520}\times 17.072$$

The ability of the extract/Pulverulentone A to inhibit pyocyanin production was tested under the same growth conditions using the relevant isolates that produced the highest amount of pigment.

Test isolates either treated with the extract/Pulverulentone A (at non-inhibitory concentrations ranging from 500 to 125 µg·mL^−1^ or 50 to 3.125 µg·mL^−1^, respectively) or untreated (positive control using DMSO, the solvent, instead of the *C. citrinus* extract/Pulverulentone-A) were grown in glycerol alanine medium for 24 h under orbital shaking at 180 rpm. Negative control was included. After incubation, pyocyanin was extracted according to the method described by Karatuna and Yagci [47], with some modifications. In brief, the cultures were centrifuged at 6000 rpm for 5 min, the supernatant was separated and an equal volume of chloroform was added with mixing. The aqueous layer was discarded and the chloroform layer (blue color) was acidified with half the volume of 0.2 M HCl. The acidified solution was vortexed and then centrifuged at 6000 rpm for 5 min. The absorbance (OD_520_) of the aqueous acidic layer (pink color) was measured using a microplate reader (BioTek Synergy 2, Winooski, VT, USA). Pyocyanin inhibition % was calculated using the following equation:

$$\mathrm{Inhibition}\;\%=\frac{\mathrm{Positive}\;{\mathrm{control}}_{\mathrm{OD}520}-{\mathrm{Test}}_{\mathrm{OD}520}}{\mathrm{Positive}\;{\mathrm{control}}_{\mathrm{OD}520}}\times 100$$

### 3.7. Effect of the Extract/Pulverulentone A on the Proteases Production

The ability of the extract/Pulverulentone A to inhibit proteases production was examined according to the method described by Vijayara-ghavan and Vincent [48]. In brief, cultures of relevant *P. aeruginosa* isolates either untreated or treated with extract/Pulverulentone A in LB broth were incubated overnight. The cultures were centrifuged at 15,000 rpm for 20 min. Then, 100 µL aliquots of the supernatants were collected and added to the wells made in 5% skim milk agar plates and incubated overnight at 37 °C. The zones of inhibition were measured and compared to the positive control.

### 3.8. Molecular Docking Study

The binding modes and affinities of Pulverulentone A, within LasR, MvfR (PqsR) and PscR receptors in *P. aeruginosa*, were predicted using molecular docking. All the molecular modeling studies were carried out using Molecular Operating Environment (MOE, 2019.0102) software. All minimizations were performed with MOE until a root mean square deviation (RMSD) gradient of 0.1 kcal∙mol^−1^Å^−1^ with MMFF94x force field and the partial charges were automatically calculated. The X-ray crystallographic structure of LasR co-crystalized with 4-bromo-2-({[(2-chlorophenyl)carbonyl]amino}methyl)-6-methylphenyl 2,4-dichlorobenzoate (TY4) (PDB ID: 3JPU) was downloaded from the protein data bank (https://www.rcsb.org/structure/3JPU). The X-ray crystallographic structure of MyfR co-crystalized with 2-[(5-nitro-1H-benzimidazol-2-yl)sulfanyl]-N-(4-phenoxyphenyl)acetamide (CZG) (PDB ID: 6B8A) was downloaded from the protein data bank (https://www.rcsb.org/structure/6B8A). The X-ray crystallographic structure of QscR co-crystalized with (2S)-2-hexyl-N-[(3S)-2-oxooxolan-3-yl]decanamide (EVY) (PDB ID: 6CBQ) was downloaded from the protein data bank (https://www.rcsb.org/structure/6CBQ). For each co-crystallized enzyme, the protein data bank was accessed on 10 April 2021.

For each co-crystallized enzyme, the water molecules and ligands that are not involved in the binding were removed, and the protein was prepared for the docking study using *Protonate 3D* protocol in MOE with default options. The co-crystalized ligands (TY4, CZG and EVY) were used to define the binding site for docking. Triangle Matcher placement method and London dG scoring function were used for docking.

### 3.9. In Vivo Cytotoxicity of Pulverulentone A Using Galleria mellonella Larvae Model

The cytotoxicity of Pulverulentone A was evaluated using the larvae of *G. mellonella* according to the method described by Allegra et al. [41]. Final instar stages of *G. mellonella* larvae were supplied by Pests and Plant Protection department, National Research Center, Egypt. Caterpillars of average weight of 200 mg were employed in the test. Cytotoxicity of Pulverulentone A was tested at concentrations of 400, 200, 100, 50, 25 and 12.5 µg·mL^−1^ prepared in sterile PBS. After disinfecting the injection site with 70% alcohol, 10 µL of each concentration were injected in the last right proleg of each group of 10 larvae using a 100-unit insulin syringe. Larvae were incubated at room temperature (25 ± 2 °C) and observed every day for 4 days for any signs of death (blackening/increased melanization with loss of movement). One group of 10 larvae was injected with 10 µL of sterile PBS and served as the negative control group and another group was not injected (quality control group). The maximum safe concentration of Pulverulentone A causing no mortality to the larvae was determined among the tested concentrations (400 to 12.5 µg·mL^−1^). The experiment was performed twice.

### 3.10. Survival of Galleria mellonella Larvae Model Infected with Non-Pretreated or Pretreated P. aeruginosa with Pulverulentone A

In order to investigate the in vivo antivirulence activity of Pulverulentone A, a *G. mellonella* larvae model as described by Peleg et al. [49] with some modifications was employed. Final instar stages of *G. mellonella* caterpillars at the same status described above were used in this assay. Gentamycin was used as drug control at sub-MIC. (Its MIC was previously determined [31] and confirmed in this study to be 1 mg·mL^−1^. In brief, twofold serial dilutions of gentamycin ranging from 4000 to 30 µg·mL^−1^ were prepared in LB broth. In a 96-well microplate, 100 µL of each concentration was added to 100 µL of an overnight culture in LB broth of PA-7 isolate adjusted to 10^6^ CFU.mL^−1^. The plate was incubated at 37 °C for 24 h. The MIC was determined by recording the lowest concentration that caused complete growth inhibition as was detected by the naked eye and was confirmed by measuring the OD at 600 nm compared to the positive control [50].) A total of 75 caterpillars were divided into 5 groups. Each included 15 caterpillars and received the respective treatment in form of injection in the last right proleg as follows:

Group I: Received 10 µL of 10^4^ CFU.mL^−1^ of *P. aeruginosa* isolate-PA-7 pre-treated with 100 µg·mL^−1^ of Pulverulentone A.

Group II: Received 10 µL of 10^4^ CFU.mL^−1^ of *P. aeruginosa* isolate-PA-7 pretreated with 250 µg·mL^−1^ of gentamycin; represents 0.25 × MIC.

Group III: Received 10 µL of 10^4^ CFU.mL^−1^ of un-pretreated *P. aeruginosa* isolate-PA-7 (positive control).

Group IV: Received 10 µL of sterile PBS solution (negative vehicle control).

Group V: Did not receive any treatment injection (quality control).

The caterpillars were incubated at room temperature as stated above and observed daily for 4 days for any signs of death. The experiment was performed twice.

### 3.11. Statistical Analysis

Statistical analysis was performed using GraphPad Prism 9.0.2 (GraphPad Software, Inc., San Diego, CA, USA).

## 4. Conclusions

Pulverulentone A could be proven as a potential scaffold for further optimization in the discovery of anti-virulence drugs. It is recommended to be considered as a lead compound in the semi-synthesis of new plant-based anti-virulent drugs. Furthermore, being recently reported as a potent MRSA anti-virulent agent, this study scientifically validates the *P. aeruginosa* anti-virulent potential of Pulverulentone A along with its *in vivo* safety. The perspective of our future studies will focus on the effect of this promising drug candidate on the quorum sensing pathways of different clinically isolated Gram-positive and Gram-negative bacteria and other microbial pathogens.

## Figures and Tables

**Table 1:** Docking results of binding of Pulverulentone A with LasR, MvfR/PqsR and QscR.

Enzyme	Binding Score (kcal/mol)	Amino Acids	Interacting Groups	Type of Interaction	Bond Length
LasR	−12.6517	Leu36	O (C=O)	H-bond acceptor	3.90
		Tyr56	O (C=O)	H-bond acceptor	3.34
		Asp73	CH	H-bond (non-conventional)	3.26
		Thr75	CH_3_	H-bond (non-conventional)	3.90
		Ser129	O (C=O)	H-bond acceptor	2.98
MyfR/PqsR	−9.2847	Leu197	O (C=O)	H-bond acceptor	3.68
		Leu207	OH	H-bond donor	2.87
		Leu208	O (C=O)	H-bond acceptor	3.76
		Ile236	CH_3_	H-bond (non-conventional)	3.82
QscR	−13.3577	Gly40	CH_3_	H-bond (non-conventional)	3.37
		Asp75	CH_3_	H-bond (non-conventional)	3.81
		Met127	OH	H-bond donor	3.31
		Ser129	CH_3_	H-bond (non-conventional)	3.72

## Data Availability

Available upon request.
